# A Case of Secondary Autoimmune Hemolytic Anemia Successfully Treated With Rituximab

**DOI:** 10.7759/cureus.62466

**Published:** 2024-06-16

**Authors:** Mary Therese Thomas, Roy L Dennis, Jacob Trimble, Zachary Bondranko

**Affiliations:** 1 Internal Medicine, Grand Strand Medical Center, Myrtle Beach, USA; 2 Internal Medicine, Edward Via College of Osteopathic Medicine (VCOM) Carolinas, Myrtle Beach, USA; 3 Family Medicine, Grand Strand Medical Center, Myrtle Beach, USA

**Keywords:** intensive and critical care, hematology disorders, ischemic gangrene, plasmapheresis treatment, rituximab therapy, cold agglutinins, autoimmune hemolytic anemia (aiha), ebv-positive

## Abstract

Secondary cold agglutinin autoimmune hemolytic anemia (AIHA) occurs most commonly due to infectious causes like *Mycoplasma pneumonia* and, more rarely, Epstein-Barr virus(EBV). Here we present a case of a 69-year-old female presenting with generalized weakness, who was found to have cold agglutinin hemolytic anemia. She unfortunately experienced some of the most severe complications of the disease including encephalopathy, hypoxia, and dry necrosis of peripheral extremities. Further investigation revealed an EBV infection, the rarest infectious cause of cold AIHA. She was started on steroids, the mainstay of treatment, but continued to worsen over the course of her extensive stay in the intensive care unit (ICU). Given the severity of the disease, the decision was made to use plasmapheresis and rituximab, the monoclonal antibody directed against CD20, as an experimental therapy. After adjunctive therapy was initiated, the patient began to clinically improve and ultimately made a full recovery. Rituximab is historically only effective in primary cold AIHA, but it appeared to elicit significant clinical improvement with our use in secondary cold AIHA. While there have been a handful of studies demonstrating its successful use in secondary cold AIHA, we propose that this medication be further studied to prevent the significant morbidity and mortality associated with the disease.

## Introduction

Autoimmune hemolytic anemia (AIHA) can be classified as either warm or cold. Warm and cold AIHA are distinguished with direct antiglobulin (Coombs) tests. Warm AIHA causes agglutination of red blood cells at temperatures above 37°C, while cold AIHA does at temperatures below 37°C [1-3]. Cold agglutinin AIHA can be further subdivided into primary and secondary diseases. Primary cold agglutinin disease uses the IgM immunoglobulin to activate the complement system and is monoclonal [4-6]. IgM functions to activate the complement system for protection against foreign bodies. The IgM-bound immunoglobulin binds to the RBCs and recruits C1, C2, and C4 [4]. These complement components lead to the production of C3 convertase which cleaves C3 into C3a and C3b [7,8]. The C3b complement components coat the RBCs phagocytosed by macrophages in the reticuloendothelial system [8]. Primary cold agglutinin AIHA can be diagnosed with the cell markers for C3b and IgM.

Secondary cold agglutinin AIHA occurs due to infectious or malignant causes. Less than 1% of all AIHA cases are caused by the Epstein-Barr virus (EBV). The secondary cold agglutinin AIHAs are polyclonal IgM mediated against the I-antigens or i-antigens [9,10]. Approximately less than 1% of the population has the i-antigen on their RBCs, making AIHA caused by EBV a very rare diagnosis [9,10].

In primary cold AIHA, the use of rituximab 375 mg weekly for four weeks and plasmapheresis have been used with much success to induce remission in those patients with primary disease [11]. Treatment for secondary cold AIHA due to EBV is supportive; the underlying viral illness is usually self-limiting. To minimize agglutination of the red blood cells, actions like keeping room temperatures high, using warming blankets, and warming intravenous fluids or blood products prior to administration are some of the most popular measures, although there is little evidence to actually support the efficacy of such measures [11]. Some evidence suggests that unless there is severe, symptomatic anemia, there should be no other interventions for treatment [11].

In this study, we present a patient with this rare finding of the IgM antibody directed against the i-antigen causing cold AIHA due to an EBV infection. The critical nature of the patient's disease course necessitated unusual and unconventional treatment with rituximab. The present case demonstrates that perhaps this agent could be used as a curative therapy in those with severe, refractory secondary cold AIHA.

## Case presentation

Our patient was a 69-year-old female with a past medical history of tobacco use and osteoarthritis who presented after 24 hours of generalized weakness. Her husband found her on the bathroom floor. She denied any head trauma or loss of consciousness. Most of the history and review of symptoms was provided by her husband, as the patient was mildly confused. The patient endorsed fatigue and chills. Otherwise, the review of systems was negative for fever, headache, vision changes, chest pain, shortness of breath, abdominal pain, nausea, vomiting, diarrhea, constipation, and focal neurological deficits.

The patient's vital signs revealed fever (103.9°F), tachycardia (HR: 106), and borderline hypotension (BP: 95/50 mmHg). Physical examination revealed a well-developed, well-nourished, but somnolent and confused female, oriented to person only. She had jaundice with a small bruise on her right hand. Her eyes revealed scleral icterus. Her abdomen was diffusely tender to palpation but worse in the right upper quadrant with moderate hepatomegaly. Bowel sounds were present in all four quadrants and there was no abdominal distention. Cardiac, pulmonary, and musculoskeletal examinations were normal. CT head without contrast revealed no acute intracranial abnormality. The patient's lab evaluation revealed multiple significant derangements, but the hepatitis panel and lipase were both negative (Table 1).

**Table 1:** Initial laboratory values for the patient upon presentation.

Variables	Patient’s values	Normal range
White blood cell	19.0 k/mm^3^	3.7-10.1 k/mm^3^
Hemoglobin	8.0 g/dL	11.6-15.4 g/dL
Mean corpuscular volume	126 fL	79.2-97.2 fL
Platelets	86 k/mm^3^	156-352 k/mm^3^
Anion gap	15 mEq/L	3-11 mEq/L
Blood urea nitrogen	46 mg/dL	7-20 mg/dL
Lactic acid	5.3 mmol/L	0.7-2.0 mmol/L
Aspartate aminotransferase	142 U/L	15-46 U/L
Alanine aminotransferase	34 U/L	13-69 U/L
Total bilirubin	28 mg/dL	0.1-1.1 mg/dL

The macrocytosis in the setting of elevated aspartate aminotransferase (AST)-to-alanine aminotransferase (ALT) ratio raised concerns for alcoholic hepatitis, but the patient and her husband adamantly denied alcohol use or a history of liver disease. Her husband stated that she was completely in her normal state and did not appear pale before hospitalization. Acetaminophen levels were within normal limits. Right upper quadrant ultrasound revealed gallbladder sludge with no evidence of acute cholecystitis and an enlarged common bile duct measuring at 4 mm.

The leading differential at the time of admission to the intensive care unit was an unidentified infection leading to severe sepsis, with subsequent jaundice from mass hemolysis. She was started on intravenous (IV) vancomycin, cefepime, and metronidazole. Her clinical condition deteriorated immediately after admission. She became lethargic and less responsive. She developed acute hypoxic respiratory failure requiring 40 L/min of high-flow nasal cannula oxygen.

Two blood culture samples grew Gram-negative bacilli one day after admission, which provided the most likely etiology of her presentation - hemolysis secondary to bacillary bacteremia. Intravenous meropenem was substituted for metronidazole and cefepime to cover for possible extended-spectrum beta-lactamase bacteria according to our local antibiogram. A peripheral blood smear was negative for schistocytes, but her labs were consistent with ongoing hemolysis. Hematology was consulted at the same hospital on day two and raised concern for possible cold agglutinin hemolytic anemia after her peripheral smear showed active agglutination requiring frequent rewarming.

**Figure 1:**
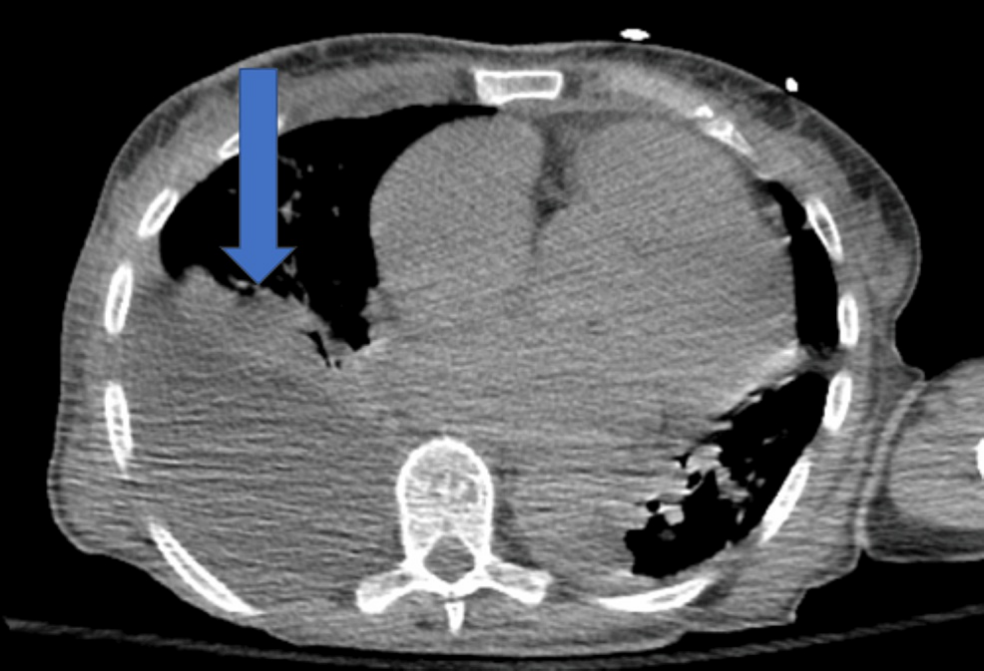
Right-sided pleural consolidation and effusion, with small left pleural effusion.

**Figure 2:**
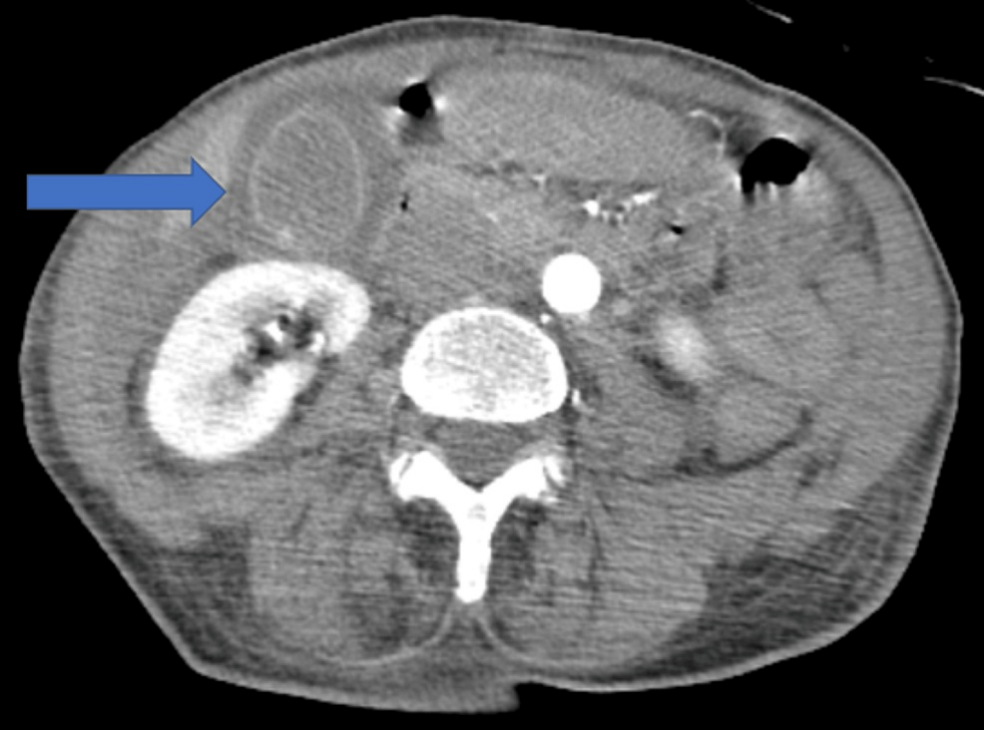
Diffuse gallbladder wall thickening.

**Figure 3:**
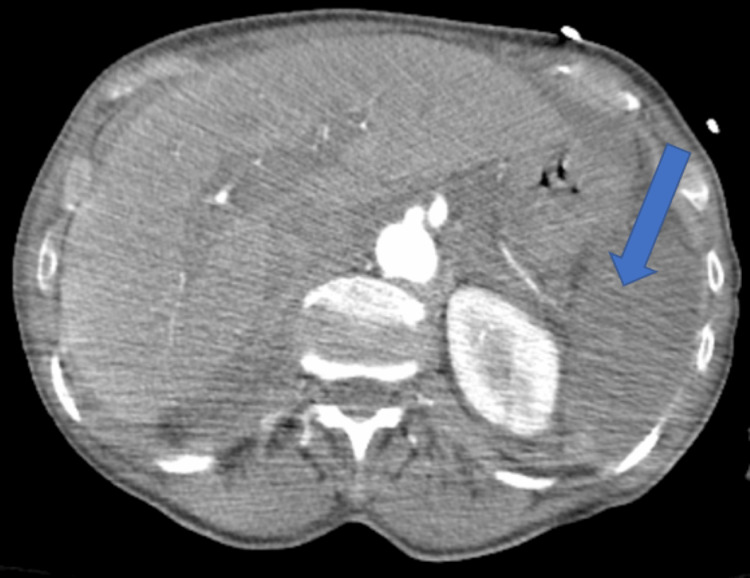
Poor perfusion through the splenic artery and hypoperfusion of the spleen.

**Figure 4:**
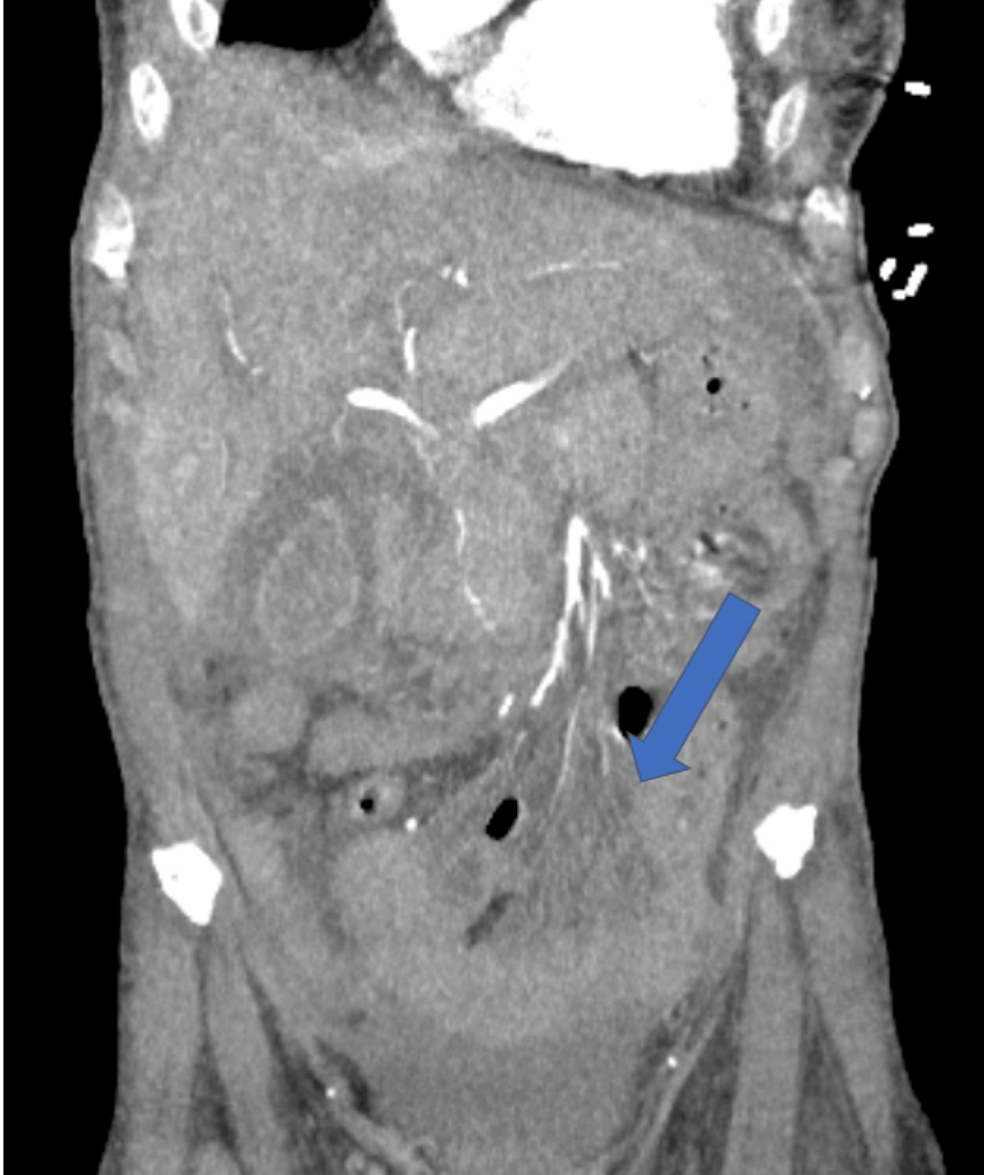
Poor perfusion through mesenteric arteries to the bowels.

Unfortunately, during the early morning of hospital day three, while undergoing a CTA of her abdomen pelvis, the patient had worsening hypoxia and required emergency intubation. A repeat CT chest showed significant worsening of bilateral opacities with a new right basilar pleural effusion and small left pleural effusion, in addition to gallbladder wall thickening and diffuse hypoenhancement of the spleen and bowel, concerning infarct/hypoperfusion (Figures 1-4). Azithromycin was initiated for atypical bacteria coverage. General surgery was consulted for possible cholecystectomy, but given her critical state and high risk of intraoperative mortality, surgery was deferred.

An EBV IgG and nuclear antigen antibody were sent for Epstein-Barr virus (EBV). On hospital day four, results returned positive. This positive outcome provided an alternate explanation for the patient's overall presentation - secondary cold agglutinin autoimmune hemolytic anemia secondary to EBV infection with concomitant Gram-negative bacillus bacteremia. Eventually, the initial “positive” blood cultures were determined to be false positives as speciation revealed a common skin contamination. This left EBV infection as the primary causative organism.

That same hospital day four, her bilirubin reached a peak of 87.9 mg/dL, with associated severe scleral icterus, jaundice, and the early signs of acrocyanosis of the most distal tips of her digits. Her condition continued to decline over the next several days despite being on antibiotics and supportive measures for cold agglutinin anemia such as heated transfusions, heated blankets, and increased room temperature. She required continuous renal replacement therapy (CRRT) due to acute renal failure suspected to be interstitial nephritis due to the EBV. During this period, the patient remained mechanically ventilated and unconscious without sedation.

After a week without improvement, hematology recommended starting plasmapheresis for a total of three treatments, followed by initiation of rituximab IV 375 mg for four doses. Despite evidence only to support its use in primary, not secondary, cold agglutinin disease, the decision was made to start this treatment given the severity of her illness. Plasmapheresis was initiated to remove persistent cold agglutinins, but due to a national distribution shortage of albumin during the COVID-19 pandemic, this process was difficult to complete. The rounds of plasmapheresis she was able to receive resulted in the extraction of black, tarry, semi-gelatinous fluid.

After her first day of treatment, the patient experienced the first improvement in her mental status since she initially lost consciousness and continued to recover with continued therapy. On day 12 of intubation, she underwent tracheostomy placement with general surgery. Her mental status continued to improve, and on hospital day 27, she was no longer encephalopathic and was able to breathe room air with her t-collar. Unfortunately, the most distal parts of her extremities, including her fingers, toes, tips of the ears, and nose had experienced significant acrocyanosis secondary to the poor perfusion. She was transferred to the general medicine floor, where she remained for approximately six weeks before being discharged home. By that time, the patient spent a total of 11 weeks in the hospital.

## Discussion

In clinical practice when presented with encephalopathy and jaundice, it is important to rule out hepatic, obstructive, and infectious causes. Obtaining appropriate imaging (i.e., ultrasound, CT abdomen, MRI, or magnetic resonance cholangiopancreatography {MRCP}) can help rule out hepatic versus obstructive causes of symptoms. In cases like ours that include newly diagnosed hemolysis, early consultation with hematology and a peripheral blood smear should be a priority to rule out hemolysis [12,13]. For a diagnosis of secondary cold AIHA, the presence of anemia and recent positive infection with a positive Coombs test confirms the diagnosis [11]. With cold agglutinin disease, the C3b complement protein marks the RBC for phagocytosis by splenic and hepatic cells, which is why there are no schistocytes seen on the smear. Elevated LDH and haptoglobin are some of the most useful and specific markers for diagnosis [14].

There are not many studies that have performed investigations into the treatment of infectious secondary cold AIHA. Usually, the level of anemia is so mild (hemoglobin >10 g/dL) and the viral course is self-limited, that many do not have a need for any treatment beyond symptom management [14,11]. Exceptions include patients with chronic pulmonary disease, coronary artery disease, or those undergoing procedures where plasma may have to undergo cooling, like cardiothoracic surgery [14]. What makes studying secondary cold AIHA difficult is the usual self-limiting nature of the disease and the limited degree to which it causes severe symptoms requiring hospitalization. Symptoms of anemia may not present until weeks after the initial infection [11]. The effects of corticosteroids have been inconclusive and, unless hemolysis is significant or there appears to be no improvement or dependence on blood transfusions develops, there is limited suggestion to their use [15,16]. Blood product transfusion can be helpful to combat symptoms, but appropriate measures such as warming of the blood during administration and examination of donor blood alloantibodies and complement levels must be taken [16].

Rituximab (a chimeric human IgG1-κ monoclonal antibody against the protein CD20) has traditionally shown effectiveness in patients with primary cold agglutinin disease and takes several weeks to work at the level of the thymus. Rituximab binds to CD20 on B-cells inducing cell death and, ultimately, suppression of B-cell mediated production of antibodies, cytokines, and their antigen-presenting cell function [17]. The effectivity yields about a 50% positive response to the anemic crisis and increases hemoglobin levels by an average of four points [14]. The inclusion of bendamustine in combination with rituximab increased responsiveness to 80-100% in those with underlying leukemia [18]. Eculizumab, the anti-C5 antibody, acts earlier on in the complement cascade to block fixation to the red blood cell surfaces and increases hemoglobin by around one unit point, reduces the level of LDH, and has been reported immediately to stop hemolysis [19]. To our knowledge, none of these medications have been tested on secondary cold AIHA.

The decision to use rituximab in this case of secondary disease was made as a "last ditch effort" in our patient, given she was not improving with traditional treatment for secondary AIHA. Meta-analyses propose that there may be some efficacy in the use of rituximab in severe, refractory disease in the acute setting of two to four months [17]. Some retrospective studies even suggest its effectiveness in the treatment of relapsing disease, calling for its use to be considered a nonsurgical alternative to splenectomy in that patient population [20]. The overall goal for the treatment of cases of secondary cold agglutinin AIHA is to prevent the progression of the disease, avoid dry necrosis of peripheral extremities, and prevent acute hypoxic emergencies, ultimately decreasing morbidity and mortality of this disease.

## Conclusions

The patient was found to have cold agglutinin autoimmune hemolytic anemia refractory to traditional steroid treatment. Usually, patients presenting with generalized fatigue, jaundice, and peripheral necrosis should have their history of recent illness reviewed for possible infectious causes like *Mycoplasma pneumoniae *or EBV infection. Understanding the clinical presentation and past medical history of patients with cold agglutinin AIHA can prevent the progression of disease and loss of peripheral extremities. A broad differential is important when being presented with hemolysis of intrinsic and extrinsic causes. Obtaining a thorough history can help narrow down the differential and give a path toward treatment plans. Most importantly, nontraditional treatment with plasmapheresis and rituximab in secondary cold AIHA may provide a new treatment option in severe or refractory cases.
